# Vitamin D Deficiency and Avascular Necrosis in Patients With Sickle Cell Disease: A Retrospective Cohort Study

**DOI:** 10.7759/cureus.72269

**Published:** 2024-10-24

**Authors:** Abdullah J Tammas, Luluh B Albehlal, Fahad Alabbas

**Affiliations:** 1 Pediatric Hematology/Oncology, Prince Sultan Medical Military City, Riyadh, SAU; 2 Epidemiology and Public Health, Prince Sultan Military Medical City, Riyadh, SAU

**Keywords:** adult, avascular necrosis, avn, children, saudi arabia, scd, sickle cell disease, vdd, vitamin d deficiency

## Abstract

Introduction

Sickle cell disease (SCD) is an inherited hemoglobinopathy with complex multi-systemic involvement. Avascular necrosis (AVN) is a devastating complication among patients with SCD. Vitamin D deficiency (VDD) is a common finding in SCD patients. However, there is a paucity of research that evaluates the potential role of VDD as a risk factor for AVN in patients with SCD. This study aimed to assess the association between VDD and AVN and determine the patterns of presentation, risk factors, diagnosis, and treatment of AVN in patients with SCD.

Methods

We conducted a retrospective cohort study of consecutive SCD patients diagnosed with VDD from January 2020 to December 2022 at Prince Sultan Medical Military City (PSMMC), Riyadh, Saudi Arabia. Inclusion criteria: (1) adults and children with confirmed SCD and (2) tested for VDD. We excluded patients who underwent bone marrow transplants. The associations were assessed using the chi-square test. Logistic regression was performed to control for confounding.

Results

The study included 711 eligible patients; 271 (38%) were children. VDD affected 301/711 (42.3%). VDD was noticed in 32.2% and 48.1% of children and adults, respectively. AVN was diagnosed in 127/711 (17.9%) patients. The mean age at AVN diagnosis was 30.9 ± 11 years. The most common presentation of AVN was chronic joint pain (> 95%). The most frequently affected joint was the hip (86%). The majority of patients (76.4%) were late in stage at diagnosis of AVN (III/IV). Patients with a history of VDD were significantly associated with a higher risk of AVN (p < 0.001). Using multivariate logistic regression, SCD patients with VDD have 9.79 times the odds of AVN compared with SCD patients without VDD (OR 9.79; 95% CI: 5.25 - 18.26). Other statistically significant risk factors for AVN included frequent vaso-occlusive crises, older age, history of acute chest syndrome, and high body mass index.

Conclusion

AVN is a significant cause of morbidity in our SCD cohort. Most of the patients had advanced AVN disease. VDD is an independent risk factor for the occurrence of AVN. Well-designed randomized trials are required to determine the effect of vitamin D supplementation in reducing AVN in patients with SCD.

## Introduction

Sickle cell diseases (SCD) are a group of inherited hemoglobinopathies with a worldwide prevalence of eight million people living with SCD. Hemoglobin S (HBS) results from a single genetic mutation in the β-globulin chain [1]. In the deoxygenated state, β-chain subunits on different HBS molecules bind together, forming HBS polymers that increase cellular rigidity and distort the red blood cells (RBC) membrane, causing RBC to assume a crescent or sickled shape. RBC sickling generates a cycle of events that perpetuate chronic hemolysis, chronic anemia, inflammation, and vaso-occlusion. Hemolytic anemia and vaso-occlusion in SCD patients underlie the bone pain episodes and end-organ damage such as cerebrovascular, cardiopulmonary, avascular necrosis (AVN), and kidney disease. Hydroxyurea, the earliest FDA-approved drug, is the most commonly used drug to manage SCD. It promotes fetal hemoglobin production and inhibits ineffective erythropoiesis. Clinically, hydroxyurea can reduce the frequency of vaso-occlusive crisis (VOC) and acute complications in SCD patients [2].

AVN, commonly known as osteonecrosis, is a devastating chronic consequence of SCD that has been well-documented in the literature [3-5]. AVN prevalence in SCD increases with age, from 3% in children under 15 to 50% by 35 [4-6]. An early study conducted in Saudi Arabia showed a significant prevalence of AVN of the femoral head (31%) [7]. AVN is believed to be a form of ischemic bone injury resulting from reduced blood flow and recurrent perfusion, ultimately leading to bone necrosis and joint collapse [8]. It predominantly affects the epiphyses of long bones, such as the femoral and humeral heads, with the femoral head being the most commonly affected due to limited blood supply [9].

Although over 50% of AVN patients are initially asymptomatic, the disease can progress to hip pain that worsens with weight bearing, an abnormal gait, and eventually joint collapse, typically within 5-10 years of diagnosis [8,10]. In a six-year study involving 2590 SCD patients, 10% developed femoral head AVN, and nearly 50% had bilateral disease [10]. The humeral head is less commonly involved than the femoral head, with a prevalence of 5.6% [11].

Advanced age, male gender, high hemoglobin, leukopenia, simultaneous alpha thalassemia, recurrent VOC, acute chest syndrome (ACS), and low bone mineral density are risk factors for AVN in SCD [4,6]. High fetal hemoglobin (HG-F) may lower AVN risk [4,5]. Age is a major risk factor, with the median age of AVN diagnosis between 28 and 40. One study suggests males are more likely to develop AVN [12].

Early diagnosis is crucial for preventing the progression of AVN in SCD, as over half of patients are asymptomatic, especially in the early stages [12]. The natural course of AVN is progressive, and MRI is the primary diagnostic modality. Without treatment (supportive care or surgical intervention), the condition will progress, leading to irreversible degenerative changes and, ultimately, joint collapse [8]. AVN can be staged using several classifications. One of the most widely used classifications is Ficat and Arlet, which divides AVN into five stages based on clinical features, MRI, and plain radiographs. Stage 0 shows normal plain radiographs and MRI without any clinical features, while Stage 4 shows degenerative changes in plain radiographs and MRI along with pain and/or limping [5,8].

Vitamin D deficiency (VDD) is estimated to affect 60-96% of SCD patients, according to several studies [13-15]. Data regarding VDD in SCD in Saudi Arabia is limited; however, one study showed that 82% of SCD patients in the Eastern province have VDD, with a higher risk seen in younger patients and patients with frequent VOC [16].

Despite the prevalence of VDD in patients with SCD, research on its contribution to the onset of AVN is limited. Furthermore, there is little data available on the prevalence of AVN in Saudi Arabia. Based on our literature review, this is the first study that has specifically evaluated the role of VDD in the development of AVN in SCD patients. The study aims to estimate AVN prevalence, evaluate the association between VDD and AVN, and determine the presentation patterns, risk factors, diagnosis, and treatment of AVN in patients with SCD.

An abstract of this article was published online during the 65th ASH annual meeting and exposition, which took place on 9-12-2023.

## Materials and methods

Study design and data source

This was a retrospective cohort study evaluating the risk of AVN in SCD patients with VDD at Prince Sultan Medical Military City (PSMMC) from January 2020 to December 2022. Patients with the following criteria were included: (1) adults and children with confirmed SCD through hemoglobin electrophoresis; (2) and tested for VDD. We excluded patients who underwent bone marrow transplants. The data were obtained from electronic health records (EHR) and included demographic features (age, gender, and BMI), SCD genotypes, the presence of other hemoglobinopathies (e.g., alpha thalassemia, glucose-6-phosphate dehydrogenase (G6PD)), hydroxyurea treatment, episodes of VOC, history of acute chest syndrome and laboratory parameters (WBC, Hemoglobin (HG), Hematocrit (HCT), Platelets (PLT), and fetal-hemoglobin (Hb-F)). The clinical presentation, age at diagnosis, site, grade, and management of AVN were also documented. The research ethics committee at PSMMC approved the study, and the study number was 1664.

Study population

The patients were divided into two distinct groups. The exposure group consisted of patients diagnosed with VDD, while the comparison group comprised patients without VDD. SCD encompasses patients with a confirmed diagnosis of SCD using electrophoresis. MRI staged and confirmed the diagnosis of AVN. The Ficat and Arlet classification was used for the purpose of staging AVN. VDD was defined as 25-hydroxyvitamin D<30 nmol/L (optimal level is ≥30 nmol/L). A mean value of three readings of laboratory parameters was taken in a steady state (no recent admission or blood transfusion within the past three months). VOC was defined as acute and severe pain requiring emergency department visits for management lasting more than 4 hours and/or hospitalization. Three or more VOCs were considered frequent VOCs. In our institution, patients aged 14 years or less are considered pediatric.

Statistical analysis

Data were analyzed using descriptive and inferential statistics via IBM SPSS® version 26 (IBM Corp., Armonk, NY). Continuous variables were presented as means±SD and medians (maximum, minimum). Categorical variables were reported as frequencies and Percentages. The chi-square test was applied to determine whether there is a statistically significant association between AVN in patients with sickle cell disease and VDD. Binary logistic regression was used to predict the presence or absence of AVN based on values of vitamin D levels along with a set of predictor variables such as age, gender, SCD genotype, frequent VOC, Acute chest syndrome (ACS) (history), fetal hemoglobin (HG-F) level, BMI which were adjusted for the confounding. Outliers were detected through Z-Score and then excluded from the analysis. All results were considered statistically significant at the level of p<0.05.

## Results

General characteristics of the cohort

The study enrolled a cohort of 711 patients who met the eligibility criteria. Four patients were excluded as they underwent bone marrow transplants. The demographic and clinical characteristics of the cohort (Table 1). The cohort median age was 20 years (range 1-62). Patients with VDD accounted for 42.3% of the total cohort. The percentage of children was found to be 38%, while males accounted for 45.9%. Among the patients included in the study, a majority of 73.6% were diagnosed with homozygous sickle cell disease (HbSS disease), while 25.8% were diagnosed with sickle beta plus thalassemia (HbS β+thal). Additionally, it was found that 75% of the recruited patients were utilizing hydroxyurea as part of their treatment regimen.

**Table 1:** Demographics and clinical characteristics of the cohort SCD: Sickle cell disease; HG: Hemoglobin; HU: Hydroxyurea; VOC: Vaso-occlusive crisis; ACS:  Acute chest syndrome; AVN: Avascular necrosis; BMI: Body mass index; VDD: Vitamin D deficiency ^a^ Median (Min-Max), ^%^ BMI for age percentile used for pediatric, ^* ^Three infants’ cases were excluded from BMI calculation due to (inapplicability/ No available data)

	VDD (301)	No-VDD (410)	All (711)	p-value
Clinical	n (%)	n (%)	n (%)	
Age ^a^	22 (3-56)	18 (1-62)	20 (1-62)	<0.001
Classification				
Pediatric	89 (29.5%)	182 (44.4%)	271 (38%)	<0.001
Adult	212 (70.5%)	228 (55.6%)	440 (62%)	
Gender				
Male	137 (45.5%)	189 (46%)	326 (45.9%)	0.879
Female	164 (54.5%)	221 (54%)	385 (54.1%)	
SCD genotypes				
HG S/S	231 (76.8%)	292 (71.2%)	523 (73.6 %)	0.226
HG S/β	69 (22.9%)	115 (28.1%)	184 (25.8 %)	
Other (2 HG S/D, 2 S/E)	1 (0.33%)	3 (0.73%)	4 (0.60%)	
HU use	224 (74.4%)	309 (75.4%)	533 (75%)	0.773
Frequent VOC	115 (38.2%)	97 (23.7%)	212 (29.8 %)	< 0.001
ACS	40 (13.3%)	32 (7.8%)	72 (10.1 %)	0.016
AVN	89 (29.6%)	38 (9.3%)	127 (17.9 %)	< 0.001
BMI ^% *^				
Underweight (< 18.5 (5%))	113 (37.5%)	199 (48.9%)	312 (44.1%)	0.010
Normal (18.5 – 24.9 (5-85%))	158 (52.5%)	163 (40.1%)	321 (45.3%)	
Overweight (25 – 29.9 (85-95%))	19 (6.3%)	27 (6.6%)	46 (6.5%)	
25 Obese (30 – 39.9 (>95%)) & 4 Extreme obesity (> 40)	11 (3.7%)	18 (4.4%)	29 (4.1%)	

AVN presentation, diagnosis, risk factors, and management

The clinical characteristics of the study population with AVN are shown in Table 2. The median age to develop AVN was 30 years (range 5-62). Furthermore, adults constituted a significant majority, accounting for around 86% of AVN cases. The study identified several risk factors that were shown to be statistically significant (P value < 0.05) for AVN. These risk factors included VDD, frequent VOC, advanced age, history of acute chest syndrome (ACS), low levels of HG-F, and high body mass index (BMI) (≥ 25 in adults and ≥ 85 percentile in pediatric), as presented in (Table 2). No statistical difference was observed between AVN and the following risk factors: gender, SCD genotypes, other hemoglobinopathies, or the use of hydroxyurea.

**Table 2:** Clinical characteristics of the study population with AVN WBC: White blood cells; HG: Hemoglobin; PLT: platelet; HG-F: Fetal Hemoglobin; HCT: Hematocrit; ACS: acute chest syndrome; AVN- Avascular necrosis; VDD- Vitamin D deficiency; G6PD: glucose-6-phosphate dehydrogenase; 5 Alpha THAL trait: Alpha thalassemia trait ^a^ Median (Min-Max), ^%^ BMI for age percentile used for pediatric, ^$^ HG S/D and S/E are in (No-AVN) group, ^*^ Three infants’ cases among No-AVN group were excluded from BMI calculation, due to (inapplicability/ No available data)

		AVN (127)	No-AVN (584)	All (711)	P-Value
Clinical		n (%)	n (%)	n (%)	
Age ^a^		30 (5 - 62)	17.5 (1 -57)	20 (1 – 62)	< 0.001
Classification					
Pediatric		18 (14.2%)	253 (43.3%)	271 (38%)	< 0.001
Adult		109 (85.8%)	331 (56.7%)	440 (62%)	
Gender					
Male		66 (52%)	260 (44.5%)	326 (45.9 %)	0.126
Female		61 (48%)	324 (55.5%)	385 (54.1 %)	
SCD genotypes					
HG S/S		102 (80.3%)	421 (72.1%)	523 (73.6 %)	0.056
Other (184 HG S/β, 2 HG S/D, 2 S/E) ^$^		25 (19.7%)	163 (27.9%)	188 (26.4 %)	
HU use		90 (70.9%)	443 (75.9%)	533 (75%)	0.239
Frequent VOC		53 (41.7%)	159 (27.3%)	212 (29.8 %)	0.001
ACS		19 (15%)	53 (9.1%)	72 (10.1 %)	0.046
VDD		89 (70.1%)	212 (36.3%)	301 (42.3 %)	< 0.001
Other hemoglobinopathy					
(37 G6PD & 5 Alpha THAL trait)		5 (3.9%)	37 (6.3%)	42 (5.9%)	0.298
None		122 (96.1%)	547 (93.7%)	669 (94.1%)	
BMI ^% *^					
Underweight (< 18.5 (5%))		41 (32.3%)	271 (46.4%)	312 (44.1 %)	< 0.001
Normal (18.5–24.9 (5-85%))		60 (47.2%)	261 (44.7%)	321 (45.3 %)	
Overweight (25–29.9 (85-95%))		17 (13.4%)	29 (5%)	46 (6.5 %)	
25 Obese (30–39.9 (>95%)) & 4 Extreme obesity(> 40)		9 (7.1%)	20 (3.4%)	29 (4.1 %)	
Laboratory		Median (Min-Max)	Median (Min-Max)	Median (Min-Max)	
WBC	10 (4-16)		9 (4-18)	9 (4-18)	0.260
HG	89 (72-118)		88 (65-120)	88 (65-120)	0.047
PLT	410 (130-770)		380 (95-900)	390 (95-900)	0.119
HG-F	11 (2-33)		15 (1-57)	14 (1-57)	< 0.001
HCT	0.260 (0.190-0.330)		0.250 (0.190-0.380)	0.250 (0.190-0.380)	0.034

Overall, no statistically significant differences were seen between the AVN presentations in adults and children. The most common AVN presentation was chronic pain (> 95%), followed by limping and limited range of motion. The study found that 59% of patients had involvement in multiple joints. Specifically, 50.4% of patients had bilateral joint involvement, 4.7% had involvement of multiple different joints, and 4% had a combination of both. The most affected joints were the hip (80%) and shoulder (15.2%). The majority of patients (76.4%) were diagnosed with AVN at a late stage (stages III and IV). Hip replacement was performed on 44 patients (34.6%); most were adults, while the remaining patients were treated with physiotherapy and observation (Table 3).

**Table 3:** Difference in AVN between pediatric and adult ^b ^ Limping and limited range of motion, ^**^ Each patient might have more than one site affected. Thus, higher total values, ^c ^ (6 Knee, 1 Sacrum) AVN- Avascular necrosis

	Pediatric AVN n (%)	Adult AVN n (%)	ALL AVN n (%)	p-value
AVN initial presentation				
Chronic pain	17 (94.4%)	104 (95.4%)	121 (95.3%)	0.858
Others ^b^	1 (5.6%)	5 (4.6%)	6 (4.7%)	
AVN (Multiple vs single)				
Multiple / Same Joint (B/L)	8 (44.4%)	56 (51.3%)	64 (50.4%)	0.400
Multiple/Different Joints	1 (5.6%)	5 (4.6%)	6 (4.7%)	
Both (B/L + Different Joint)	2 (11.1%)	3 (2.8%)	5 (4%)	
Single Joint	7 (38.9%)	45 (41.3%)	52 (40.9%)	
Laterality				
Unilateral	8 (44.4%)	48 (44.1%)	56 (44.1%)	0.974
Bilateral	10 (55.6%)	61 (55.9%)	71 (55.9%)	
AVN stage				
Early (I, II)	4 (22.2%)	26 (23.9%)	30 (23.6%)	0.880
Late (III, IV)	14 (77.8%)	83 (76.1%)	97 (76.4%)	
Management				
Observation/Physiotherapy	17 (94.4%)	66 (60.6%)	83 (65.4%)	0.005
Hip replacement	1 (5.6%)	43 (39.4%)	44 (34.6%)	
Total	18 (14.2%)	109 (85.8%)	127 (100%)	
AVN Sites **	Pediatric AVN n (%)	Adult AVN n (%)	ALL AVN n (%)	p-value
Hip	17 (85%)	93 (78.8%)	110 (79.7%)	0.778
Shoulder	2 (10%)	19 (16.1%)	21 (15.2%)	
Others ^c^	1 (5%)	6 (5.1%%)	7 (5.1%)	
Total	20 (14.5%)	118 (85.5%)	138 (100%)	

Association between AVN and VDD

The study found that the prevalence of VDD was 42.3%, with a higher prevalence observed in adults (48.1%) compared to children (32.2%). Additionally, the prevalence of AVN was 17.9%, affecting a larger proportion of adults (24.8%) compared to children (6.6%). Individuals diagnosed with VDD had a higher likelihood of developing AVN compared to those without VDD, as indicated in (Table 1). A chi-square test of independence was performed to examine the relation between VDD and developing AVN. Among all age groups, the relation between these variables was statistically significant (p-value<0.001). Binary logistic regression was used to examine whether VDD was associated with the likelihood of having an AVN diagnosis (Table 4). A multivariate regression was conducted to examine the association between having AVN and VDD, which were adjusted for possible confounding factors such as age, gender, frequent VOC, history of acute chest syndrome (ACS), HG-F level, BMI, and SCD Genotype. The model was statistically significant (p-value < 0.001), suggesting that it could distinguish between those with and without an AVN diagnosis. The model explained between 25.3% (Cox & Snell R square) and 43.8% (Nagelkerke R square) of the variance in the dependent variable and correctly classified 87% of cases. As shown in Table 5, VDD significantly contributed to the model. The odds ratio corresponding to VDD (95% confidence interval) is 9.79 (5.25, 18.26). Thus, VDD is an independent risk factor for AVN.

**Table 4:** Logistic Regression Predicting the likelihood of having AVN VDD: Vitamin D deficiency

	B	SE	Wald	df	P	OR	95% CI OR	
							Lower	Upper
VDD	2.28	0.318	51.5	1	< 0.001	9.79	5.25	18.26

Subgroup analysis

The subgroup analysis investigating the relation between VDD and the development of AVN in both children and adults yielded statistically significant results (p-value < 0.008). These findings suggest that individuals with VDD, regardless of age, have a higher likelihood of developing AVN compared to those without VDD (Tables 5-6).

**Table 5:** Association between AVN and VDD among pediatric patients AVN: Avascular necrosis; VDD: Vitamin D deficiency

	Pediatric (n=-271 (38.1%))			
	AVN	Non-AVN	Total	p-value
VDD	11 (61.1%)	78 (30.8%)	89 (32.8%)	0.008
Non-VDD	7 (38.9%)	175 (69.2%)	182 (67.2%)	
Total	18 (100%)	253 (100%)	271 (100%)	

**Table 6:** Association between AVN and VDD among adult patients AVN: Avascular necrosis; VDD: Vitamin D deficiency

	Adult (n=440 (61.9%))			
	AVN	Non-AVN	Total	p-value
VDD	78 (71.6%)	134 (40.5%)	212 (48.2%)	< 0.001
None-VDD	31 (28.4%)	197 (59.5%)	228 (51.8%)	
Total	109 (100%)	331 (100%)	440 (100%)	

## Discussion

AVN is a serious complication of sickle cell disease. It can have a significant impact on the patient's quality of life. To mitigate this impact, we need to early identify and manage those patients with risk factors predisposing them to develop AVN. The primary objective of this retrospective cohort study was to investigate VDD's possible role in the development of AVN in patients with SCD. The prevalence of AVN and VDD in our cohort was 17.9% and 42.3 %, respectively. VDD was identified as an independent risk factor for the development of AVN. Other risk factors for AVN include older age, frequent VOC, history of ACS, low HG-F level, and high BMI. Chronic joint pain was the most common presentation of AVN. The majority of the patients diagnosed with AVN are in the late stages. Hip replacement was performed in more than a third of the patients.

AVN prevalence in our cohort was higher in adults (24.8%) than in children (6.6%). Multiple local studies found a higher prevalence, ranging from 25% to 31% [7,17]. These local studies had a limited sample size, were outdated, and were carried out before introducing hydroxyurea. A recent study estimated AVN prevalence among adults with SCD at 7.3% [18]. There is no local estimation of AVN prevalence in children with SCD, apart from a study with a small sample size (20 patients) that estimated AVN prevalence in children with SCD at 28.6% [19]. Globally, different studies estimated AVN prevalence between 3% in children and 50% in adults aged 35 years old [5, 6, 20]. Adesina O et al. showed a similar prevalence of AVN in adults (22%) [21].

Risk factors for AVN in our sample are similar to data in the literature and include older age, frequent VOC, and history of ACS [20-22]. The study found that AVN risk increases with age, with adults accounting for 86% of cases. The median age at AVN diagnosis in our cohort was 30 years, which is similar to several studies showing the median age between 27 and 40 years old [4,12,21]. However, AVN can occur in early childhood, with three of our patients developing AVN before eight years old [5]. Patients with HGSS genotype with concurrent α-thalassemia trait have been reported to be at higher risk for developing AVN [11,12]. Because co-inheritance of the α-thalassemia trait is uncommon in the Benin haplotype, which is mostly found in the Southwestern Kingdom and includes most of our cohort, none of our AVN patients had the α-thalassemia trait. Our study showed no significant difference in AVN prevalence based on gender or SCD genotype. We are reporting for the first time that SCD patients with a high BMI are at higher risk of developing AVN. A high HG-F level (> 10) has a protective benefit against VOC, ACS, and AVN [23,24]. Other laboratory risk factors like high hemoglobin/HCT and low MCV have been linked with AVN [11]. Our analysis supports the claim that high HG-F is a protective factor for AVN and that patients with a high hemoglobin level are at a higher risk of developing AVN [4].

The majority of our patients presented with chronic pain, with the hip being the most commonly affected joint (80%), followed by the shoulder (15%). There was no significant difference in AVN presentation based on age, apart from the shoulder being more common in adults. Our finding confirms the fact that the femoral head is the most common site of AVN and that the majority present with chronic pain [8,9]. However, we report a higher prevalence of shoulder AVN than reported in the literature [11], possibly due to the higher number of adults in our sample and the easy accessibility of MRI in our center. More than half of all age groups (55%) developed bilateral joint involvement (mainly hip), which is in line with previous studies [5,12].

The study found that 76.4% of patients were diagnosed at late stages of AVN, similar to previous studies [25,26]. This finding indicates the need to educate patients and physicians on the importance of early diagnosis and the necessity of creating a screening program to identify AVN early. Early-stage AVN prevalence was 23.6%, which is comparable to Mohsen et al. (20.5%), higher than Akinyoola et al. (18.2%), and lower than Mukisi et al. (30%) [25-27]. This variation is partly due to the diagnostic modality, where most underdeveloped countries use plain radiology, which may not be sensitive enough to diagnose early-stage AVN in contrast to MRI. About 35% of patients underwent hip replacement, with the majority being adults. This finding is higher than what was reported by Mohsen et al., where only 10% were offered a hip replacement [27].

Vitamin D is crucial in bone mineralization and calcium and phosphate homeostasis. Nearly one billion people worldwide suffer from VDD, with an estimated prevalence of 60% worldwide [13]. Its optimal level is 25-hydroxyvitamin D > 30 ng/mL. In Saudi Arabia, 40-90% of all age groups had VDD [14]. The overall prevalence of VDD in our cohort was 42.3%, with a higher prevalence in adults (48.1%) than in children (32.2%). Global and local studies showed a 60-80% prevalence of VDD in SCD patients [13,16]. The low prevalence of VDD in SCD children could be explained by the routine use of multivitamins and/or vitamin D supplements by the family or caregiver. In the present study, SCD patients with VDD had a higher risk of developing frequent VOC, ACS, and AVN. The association between VDD and frequent VOC was reported in a previous study [16]. The role of VDD in developing ACS or AVN was not previously reported in the literature. A clinical trial found that two years of vitamin D supplementation significantly reduced the rates of annual respiratory complications (including ACS) in children with SCD [28]. This finding may support the role of VDD in the development of ACS in SCD.

Limited research specifically investigates the association between VDD and AVN in SCD patients. One study conducted on patients with SCD reported no significant association between VDD and AVN, whereas another study involving pediatric patients with lupus erythematous found evidence suggesting a relationship between these two variables [7,29]. Our study found that VDD is an independent risk factor for AVN and that patients with VDD are nearly ten times more likely to develop the disease, hence suggesting a potential preventive role for vitamin D supplementation in the development of AVN.

Study limitations

In our study, the interval between VDD testing and the diagnosis of AVN was variable, and it was possible for some patients to be diagnosed with VDD after developing AVN. This study only includes patients with MRI-confirmed AVN, which may exclude those with AVN verified by plain radiographic features who never had the opportunity to undergo an MRI. Also, the outcome of hip replacements was not reported due to the unavailability of data.

## Conclusions

AVN is a devastating complication for SCD patients with modifiable risk factors. Our study showed that VDD is an independent risk factor for developing AVN. The femoral head is the most common site of AVN. Most of the patients had advanced AVN disease, and almost half developed bilateral disease. Overweight and obesity were associated with a higher risk of developing AVN. Early diagnosis and modification of risk factors are crucial. Well-designed randomized trials are required to determine the effect of vitamin D supplementation in reducing AVN in children and adults with SCD.
